# Mucosa-Associated Lymphoid Tissue Lymphoma Masked as Gastric Varices With Acute Upper Gastrointestinal Bleeding: A Case Report

**DOI:** 10.7759/cureus.26424

**Published:** 2022-06-29

**Authors:** David E Jonason, Michael Linden, Guru Trikudanathan

**Affiliations:** 1 Internal Medicine, University of Minnesota Medical Center, Minneapolis, USA; 2 Laboratory Medicine and Pathology, University of Minnesota Medical Center, Minneapolis, USA; 3 Gastroenterology, University of Minnesota Medical Center, Minneapolis, USA

**Keywords:** doppler endoscopic ultrasound, mucosa-associated lymphoid tissue (malt) lymphoma, upper gastro-intestinal bleed, subepithelial lesion, gastric varices

## Abstract

Extra-nodal marginal zone lymphoma of mucosa-associated lymphoid tissue (MALT) is uncommon and difficult to diagnose due to varied clinical presentations and endoscopic appearances masquerading as other pathology. Rarely, it has been associated with acute upper gastrointestinal (GI) bleeding. We report on a 60-year-old male who presented with an acute upper GI bleed and endoscopic findings suggestive of isolated gastric varices (GV), ultimately determined to be MALT lymphoma. Complete remission was achieved with radiation therapy, with no recurrence at a 12-month follow-up. This case highlights a unique clinical and endoscopic presentation of MALT lymphoma which providers should be aware of. We emphasize the use of endoscopic ultrasound (EUS) evaluation for accurate diagnosis.

## Introduction

Primary gastric lymphoma comprises 4-20% of all non-Hodgkin's lymphomas (NHLs) and 2-8% of primary gastric malignancies [1]. Histologically, it is categorized predominantly into intermediate to high-grade diffuse large B-cell lymphoma (DLBCL) and low-grade B-cell lymphoma of mucosa-associated lymphoid tissue (MALT). MALT lymphoma accounts for 40% of these cases and most frequently involves the stomach but can occur anywhere in the digestive system, salivary glands, skin, lungs, thyroid, breast, and liver [2,3]. The prevalence of gastric MALT lymphoma is increasing. Almost 90% is related to *Helicobacter pylori* infection [4]. Diagnosis is challenging due to vague clinical presentations and diverse endoscopic appearances [5]. Anemia from chronic or occult gastrointestinal (GI) bleeding is occasionally observed, though acute bleeding is uncommon. Early diagnosis is important to prevent these complications and disease progression.

We report a case of a 60-year-old male presenting with hypovolemic shock and acute blood loss anemia thought to be from gastric varices (GV), later confirmed to be *H. pylori*-negative MALT lymphoma. We discuss various presentations of gastric MALT lymphoma and highlight the use of endoscopic ultrasound (EUS) to assist in diagnosis and treatment.

## Case presentation

**Figure 1:**
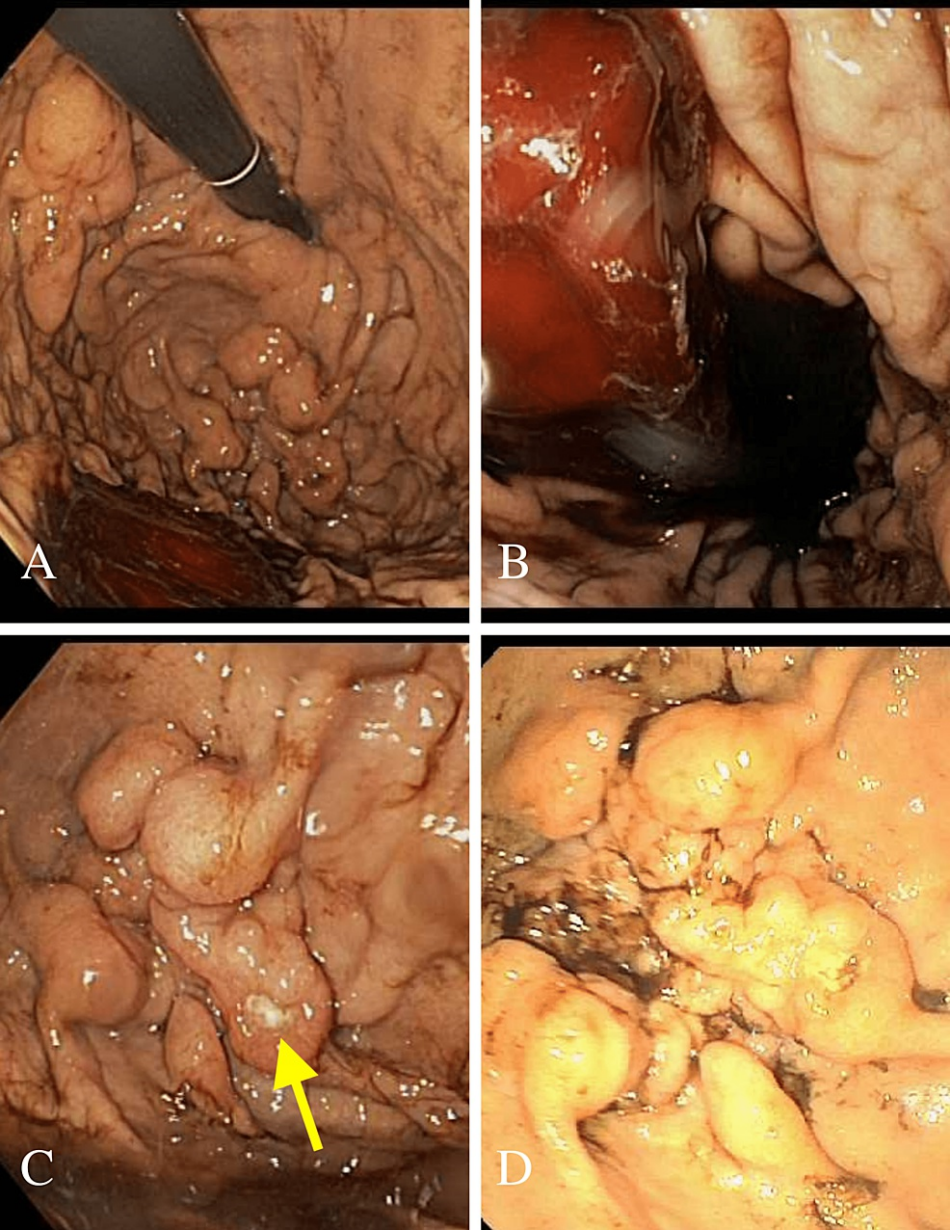
Esophagogastroduodenoscopy evaluation of gastric fundus and body (A) Bright blood in the fundus seen on EGD; (B) large blood clot in gastric body; (C) & (D) multiple prominent gastric folds in the fundus mimicking the appearance of GV. A centralized white fibrin plug versus ulcer is also seen (arrow).

A 60-year-old male with a history of occasional alcohol use (two drinks per week), tobacco use (18 pack-years), and a family history of gastric ulcers but no malignancy presented with two weeks of fatigue and a pre-syncopal episode one day prior to admission. He started naproxen for back pain and aspirin for a nonocclusive cerebral venous sinus thrombus three weeks prior. He denied abdominal pain, nausea, vomiting, or GI bleeding. A physical exam revealed tachycardia to 132 bpm (beats per minute), hypotension (90/64 mm Hg), and a hemoglobin of 6.2 g/dL, down from a 12 g/dL baseline. Intravenous (IV) fluids, three units of packed red blood cells, and IV pantoprazole were given. A computed tomography (CT) scan with contrast was remarkable for gastric wall thickening only, though a decompressed stomach limited evaluation. Esophagogastroduodenoscopy (EGD) found blood in the gastric fundus and body with suspicious fundal gastric varices and a white nipple sign (Figure 1a-1d). IV octreotide was started and he was referred for EUS-guided gluing and coiling. There was no other clinical or CT evidence of gastric varices, cirrhosis, or portal hypertension.

**Figure 2:**
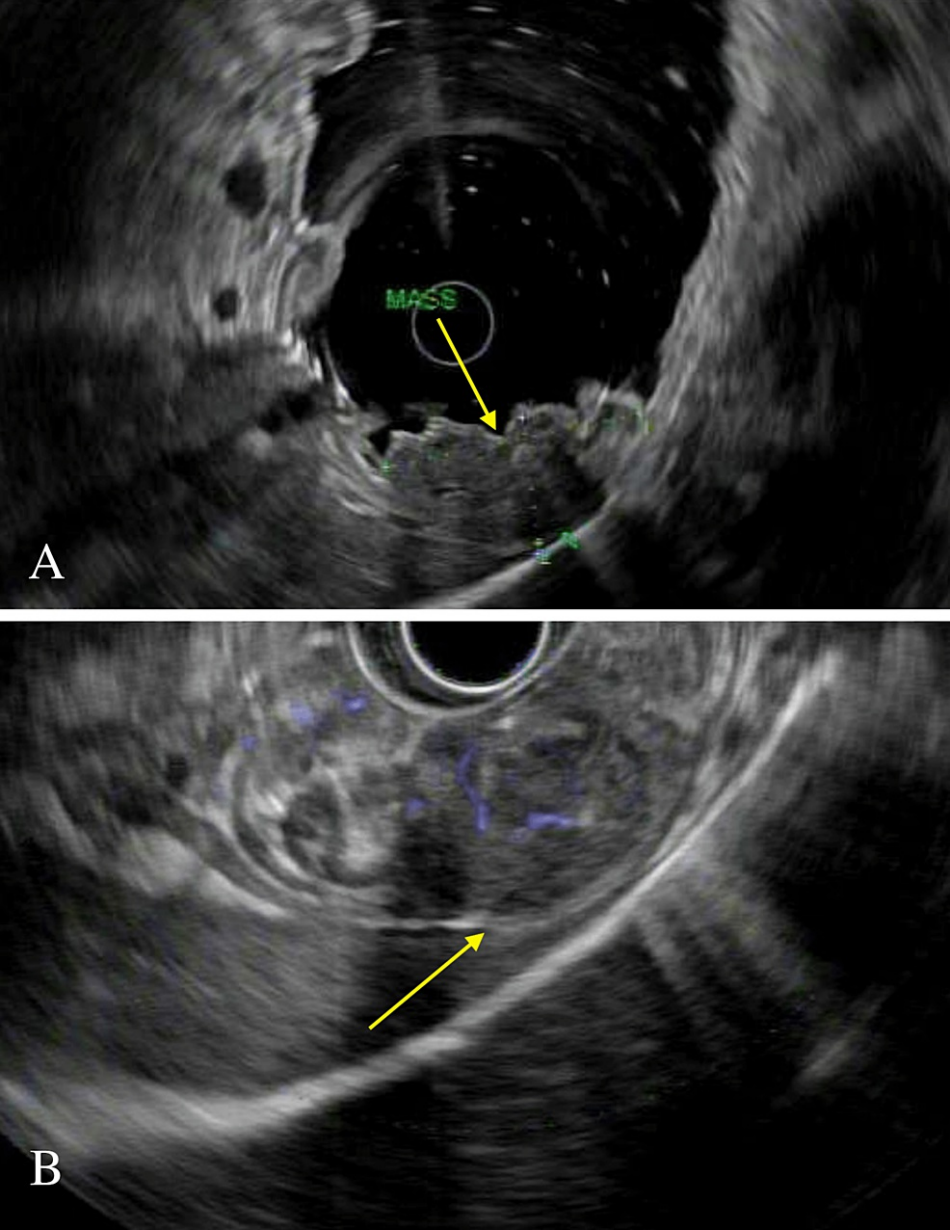
Endoscopic ultrasound showing a fundal subepithelial lesion (A) EUS showing an isolated hypoechoic mass in the fundus without invasion of adjacent serosa, spleen, or diaphragm and (B) Doppler evaluation without evidence of blood flow, excludes varix.

**Figure 3:**
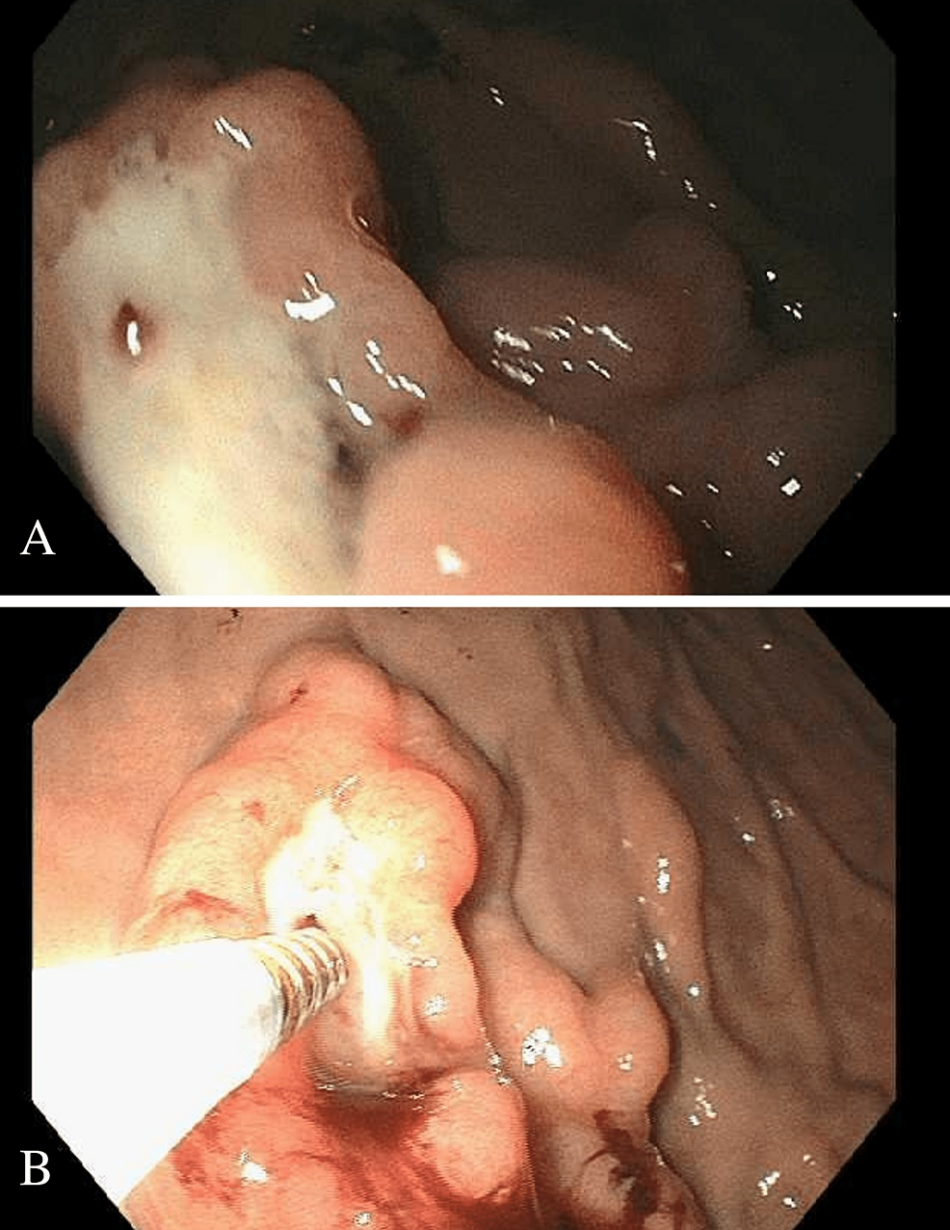
Subepithelial lesion in the gastric body (A) SEL with ulceration and a visible vessel in proximal gastric body and (B) bleeding of vessel after bipolar cauterization attempted.

**Figure 4:**
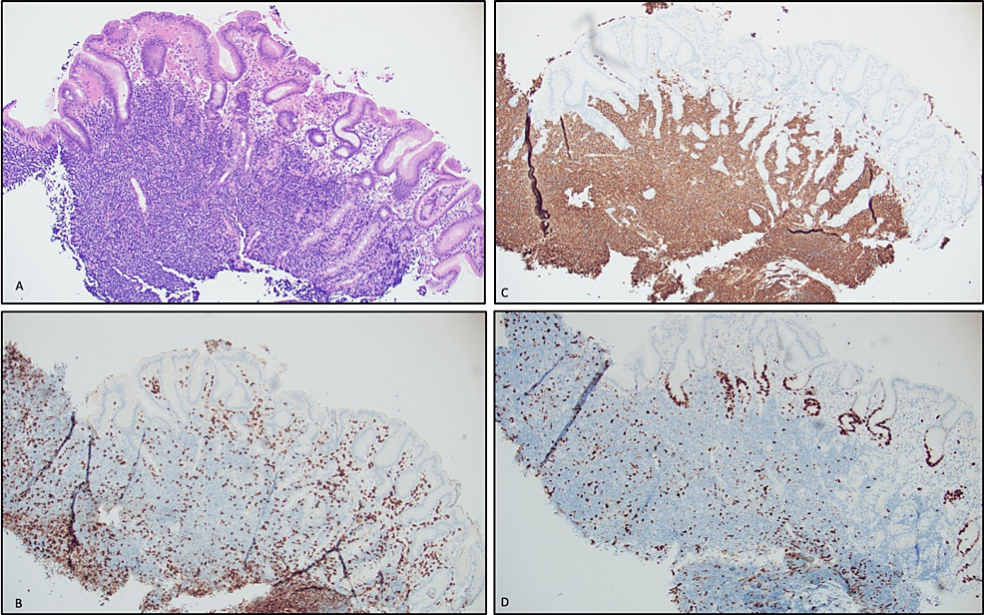
Gastric ulcer biopsy at 4× magnification (A) Hematoxylin and Eosin (H&E) stain shows a dense lymphoid infiltrate composed of small lymphocytes overrunning the lamina propria (LP); (B) IHC confirms the presence of rare, scattered CD3 positive T cells. (C) IHC demonstrating an abundance of CD20-positive B cells within the LP; (D) Ki-67 stain shows a low proliferative index confirming morphological impression of low-grade lymphoma.

EUS revealed an ulcerated subepithelial lesion (SEL) in the fundus. Sonographically, it was hypoechoic, measured 4.7 cm × 2.7 cm, and originated from the intramural wall; the exact layer was indeterminate. The outer borders were well defined with an intact interface between the mass, serosa, spleen, and diaphragm. Color Doppler failed to show vascular flow, excluding varix (Figure 2a-2b). The ulcer edge was biopsied with cold forceps. EUS-guided fine needle aspiration (FNA) with a 22-gauge ultrasound biopsy needle was also performed. Two more SELs were found along the posterior wall of the proximal gastric body and were similarly biopsied. One contained deep ulceration and a visible vessel. Bipolar cauterization of the vessel led to bleeding (Figure 3a-3b). Hemostasis was achieved with submucosal injection of dilute epinephrine (1:10,000) and three hemoclips placed under endoscopic guidance. FNA was nondiagnostic with scant cellularity. However, surgical pathology from cold forceps biopsies revealed a dense lymphoid infiltrate composed of small lymphocytes, overrunning the lamina propria (Figure 4a). Lymphoepithelial lesions were present. Immunohistochemistry (IHC) showed that the majority of the neoplastic cells were CD20 positive B cells, admixed with rare scattered CD3 positive T cells (Figure 4b-4c). The B cells lacked CD5 and CD10 and were negative for Cyclin D1, excluding mantle cell lymphoma. The findings were consistent with low-grade B cell lymphoma - extra-nodal marginal zone lymphoma of mucosa-associated lymphoid tissue (MALT). *H. pylori* antigen testing and IHC staining were negative. A positron emission tomography (PET) scan and bone marrow biopsy confirmed stage IA (localized) disease. He completed 20 fractions of gastric radiation and there has been no disease recurrence on 12-month follow-up endoscopic biopsies.

## Discussion

Gastric MALT lymphoma is often indolent and discovered incidentally on endoscopy performed otherwise. Symptoms usually do not correlate with endoscopic observations until the disease is advanced, causing bulky adenopathy, perforation, or bleeding. Patients may be asymptomatic or report vague gastrointestinal symptoms such as epigastric pain, dyspepsia, nausea, or poor appetite. Typical "B-symptoms" like fevers and night sweats are uncommon, and alarming symptoms like persistent vomiting, weight loss, hematemesis/melena, and anemia are absent in up to 70% of patients with the low-grade disease [6]. Only 15.6% of cases have been reported with upper GI bleeding [7]. Life-threatening acute bleeds are rare and seldom described in a few case reports [5,8,9].

The endoscopic appearance of gastric MALT lymphoma is variable. Commonly recognized pattern classifications include exophytic, hypertrophic with giant folds, ulcerative, petechial, normal/hyperemic mucosa, and mixed-typed [10,11]. Ulcerative patterns represent 40-50% of cases [11]. Low-grade neoplasms appear more like petechial hemorrhage or normal/hyperemic mucosa and high-grade as exophytic or ulcerated [12]. Tumors associated with *H. pylori* are often ulcerative as well. Most cases occur in the antrum, though any location in the stomach is possible [6,13].

Diverse endoscopic appearances make it hard to distinguish MALT lymphoma from gastritis, erosions, benign gastric ulcers, and other gastric malignancies [11]. Presentation as a bulky subepithelial tumor (SET) may also mimic gastrointestinal stromal tumors (GIST) or gastric leiomyosarcomas [14]. In our patient, it masqueraded as GV. Although prominent gastric folds are a known pattern of MALT lymphoma, their atypical location in the fundus and with what appeared to be a white fibrin plug after massive GI bleeding was convincing for GV. These diagnoses can be difficult to distinguish with endoscopy alone, particularly in the setting of bleeding. The lack of other clinical or CT evidence of liver disease, portal hypertension, or varices is what prompted the EUS investigation and the correct diagnosis in this case.

Direct-visualization EGD has a diagnostic accuracy of 11-22% in appropriately discriminating gastric MALT lymphoma from other disease processes based on appearance alone. This improves to 50-75% when biopsies are taken, but is still low and subject to variability depending on what part of the lesion is biopsied [11,15]. While associated endoscopic patterns exist, up to 20% of cases may have normal gastric mucosa [11]. Nothing is pathognomonic and any abnormal mucosa appreciated on endoscopic evaluation warrants further investigation. Magnifying endoscopy with narrow band imaging (NBI) has been used to improve endoscopic diagnosis by detecting changes in vasculature and gastric pits associated with MALT lymphoma [12]. However, this technique is limited to advanced specialized centers with experienced endoscopists and is primarily useful in detecting small lesions. Changes are often unrecognized in deep SELs. Larger studies are needed to evaluate the true efficacy of this technique. 

EUS serves as a multipurpose tool in gastric MALT lymphoma evaluation. Its diagnostic accuracy is 95% and it can reliably determine the number of gastric wall layers involved with a sensitivity of 80-99% and specificity near 100% [11,16,17]. The involvement of lymph nodes is also visualized. It assists in disease grading and staging, helps predict remission response to *H. pylori* eradication therapy, and tracks recurrence [18-20]. It is also used to guide FNA. As was demonstrated in this case, Doppler interrogation additionally helps detect underlying vasculature and accurately differentiate SELs from varices. It is undoubtedly an essential tool in the diagnosis and management of gastric MALT lymphoma, and it should be used to help differentiate whenever a subepithelial lesion or gastric varix is suspected.

## Conclusions

MALT lymphoma is notoriously difficult to diagnose, with varied clinical and endoscopic presentations. Massive upper GI bleeding is rare but can occur, as was demonstrated in our case. Its diverse endoscopic appearances may masquerade as alternative diagnoses, including gastric varices that are difficult to distinguish with direct visualization endoscopy alone. EUS evaluation has become an essential tool to help reliably diagnose, grade, stage, guide treatment, and monitor for relapse. Providers should have a low threshold to evaluate abnormal gastric mucosa further with EUS whenever MALT lymphoma is a possibility.
